# Evaluation of contrast sensitivity measurements after retrobulbar optic neuritis in Multiple Sclerosis

**DOI:** 10.1007/s00417-014-2590-x

**Published:** 2014-02-28

**Authors:** Marta Owidzka, Michal Wilczynski, Wojciech Omulecki

**Affiliations:** Department of Ophthalmology, Medical University of Lodz, Medical University Barlicki Hospital No.1, Kopcinskiego str. 22, 90-153 Lodz, Poland

**Keywords:** Contrast sensitivity, Multiple sclerosis, Retrobulbar optic neuritis

## Abstract

**Background:**

The evaluation of contrast sensitivity is an important additional examination that allows the physician to achieve the full picture of a patient's quality of vision. In low-contrast conditions, more discrete visual dysfunctions may be revealed, which could be overlooked in high-contrast tests.

**Methods:**

The examined group consisted of 33 eyes of 27 patients with multiple sclerosis. The study included patients with full or almost full visual acuity, without visual field defects or any other ophthalmic condition, and who had never undergone any ocular surgery or trauma. The reference group consisted of 49 eyes of 37 patients. This group included healthy subjects with full visual acuity. Contrast sensitivity was examined with a Functional Vision Analyzer™ device in photopic conditions (with and without glare) and in mesopic conditions (with and without glare).

**Results:**

In patients with multiple sclerosis who had experienced optic neuritis, contrast sensitivity was found to be significantly reduced in all spatial frequencies in both mesopic and photopic conditions (with and without glare).

**Conclusions:**

Contrast sensitivity in patients with multiple sclerosis who have also had optic neuritis is significantly reduced. This may explain patients' complaints regarding their quality of vision, despite good visual acuity. Contrastometry is a useful basis for further examination, providing additional information regarding a patient's quality of vision.

## Introduction

The evaluation of contrast sensitivity is an important examination that allows us to recognize many visual disfunctions in their early stages and to achieve the full picture of a patient's quality of vision [1–3]. In various diseases, the ability to discern low-contrast objects may be decreased, while the ability to recognize high-contrast objects may be unaffected.

During a routine ophthalmic examination, the evaluation of visual acuity with Snellen or logMAR charts is one of basic tests performed to examine visual functions. However, it is necessary to be aware of the limitations of examinations using these charts, as they are designed with high-contrast optotypes [4–7].

In low-contrast conditions, more discrete visual dysfunctions may be revealed that could be otherwise overlooked in high-contrast tests [5, 8–15]. The evaluation of contrast sensitivity is a subjective measurement of visual potential and provides information about the quality of a patient's vision [3, 15].

In most life situations, we have contact with objects of lower contrast than those on Snellen charts. For this reason, the evaluation of contrast sensitivity allows us to understand why among people with similar visual acuity, some patients complain of decreased vision and visual functioning in everyday life [13, 14, 16–19].

Contrastometry is used mainly to diagnose patients with ophthalmic diseases or patients who have undergone refractive or cataract surgery, but it is also useful in the examination of patients with neurological diseases, general medical conditions, neurotoxical disorders, and of some healthy people, in whom it is important to evaluate visual quality (e.g., pilots, drivers, etc.).

Demyelinization in the course of multiple sclerosis (MS) is one of the most frequent causes of optic neuritis. It is estimated that about 15–20 % of patients with multiple sclerosis are diagnosed with optic neuritis in the course of the disease [1, 20]. There are articles suggesting that this proportion is much higher and may be as high as 50 % [1, 21, 22]. The main symptoms include: subacute, unilateral, and decreased visual acuity, which may be accompanied by dyschromatopsy, decreased contrast sensitivity, phosphenes (positive visual phenomena) seen as white or colorful flashes, as well as ocular pain or pain around the eyeball, which increases with eye movement and afferent pupillary defect. In addition, visual field defects or, more frequently, generalized decreased retinal sensitivity may be present. Decreased contrast sensitivity is present in about 60–80 % of patients with MS; nevertheless, some of them have normal visual acuity [23–25].

The purpose of our study was to evaluate contrast sensitivity in patients with multiple sclerosis who had also suffered from optic neuritis, as well as to estimate the clinical usefulness of the Functional Vision Analyzer™ in the diagnostics of these patients.

## Methods

The examined group consisted of 33 eyes of 27 patients (10 men and 17 women), aged from 19 to 48 years old (mean 32 years), diagnosed with multiple sclerosis. The study included only patients with good best-corrected visual acuity (BCVA), ranging from 0.7 to 1.0 (mean BCVA = 0.97) without visual field defects or any other ophthalmic condition. Other inclusion criteria were: the lack of any visual symptoms, no ocular disease, no previous ocular surgery or trauma, as well as the absence of any general medical conditions. In cases when the patient experienced optic neuritis unilaterally, only one eye was included in the study. The duration of multiple sclerosis ranged from 1 to 16 years. The time between the episode of optic neuritis and our contrast sensitivity measurement exceeded 6 months in all cases.

The reference group consisted of 49 eyes of 37 patients (16 women and 21 men), aged from 20 to 81 years old (mean 43 years). This group included healthy subjects, with full visual acuity, who had never been diagnosed with any ophthalmic disease.

The analyzed data were gathered prospectively from a non-randomized consecutive series of patients. For all study protocols, we followed all tenets of the Declaration of Helsinki. All patients gave an informed consent to participate in the study. Before the study, the Ethics Committee approval was obtained (number RNN/53/11/KE).

All patients had a full ophthalmic examination before contrastometry was performed. Best-corrected visual acuity was examined with standard Snellen charts (numeral optotypes). Slit-lamp examination of the anterior segment and the eye fundus was performed. Intraocular pressure was measured with an applanation tonometer. In addition, retinal nerve fiber layer (RNFL) thickness was measured using optical coherence tomography (OCT).

In all patients, contrast sensitivity was examined with a Functional Vision Analyzer™ (Stereo Optical Co., Inc.) device in photopic conditions (with and without glare) and in mesopic conditions (with and without glare). In all patients, contrastometry was performed in the same lightning conditions, with a "distance examination strategy". Patients who had a refractive error were examined with appropriate correction.

We also evaluated vision (with and without glare, 1 lux) and day vision (with and without glare, 10 lux) in each patient, for each eye separately. Contrast sensitivity was measured for the following spatial frequencies: 1.5, 3, 6, 12, and 18 cpd (cycles per degree).

## Results

The results of contrast sensitivity measurements show that there is a significant difference in the mean contrast sensitivity between group of patients after optic neuritis in multiple sclerosis and healthy subjects (Table 1). Furthermore, in patients with multiple sclerosis, contrast sensitivity is significantly reduced in all spatial frequencies, both in photopic conditions (with and without glare) and in mesopic conditions (with and without glare) (Table 2).

**Table 1:** Comparison of contrast sensitivity measurements in various conditions, in patients after optic neuritis with the control group

Examination	Spatial frequency	Group				Z test	Significance p
		MS with optic neuritis		Control group			
		x	SD	x	SD		
Night testing without glare	1.5	**31.9**	12.1	**56.0**	25.1	4.515	**p < 0.001**
	3	**70.4**	32.9	**104.3**	34.7	3.990	**p < 0.001**
	6	**34.3**	23.4	**69.3**	33.5	4.577	**p < 0.001**
	12	**8.45**	7.52	**21.4**	16.4	4.174	**p < 0.001**
	18	**0.91**	2.50	**4.76**	4.48	4.005	**p < 0.001**
Night testing with glare	1.5	**39.9**	19.2	**65.7**	23.9	4.477	**p < 0.001**
	3	**72.1**	34.2	**109.0**	31.2	4.298	**p < 0.001**
	6	**35.9**	27.7	**68.4**	30.8	4.709	**p < 0.001**
	12	**9.79**	8.34	**20.9**	15.5	3.744	**p < 0.001**
	18	**0.61**	1.97	**4.82**	5.13	4.094	**p < 0.001**
Day testing without glare	1.5	**28.6**	12.7	**50.4**	19.3	5.238	**p < 0.001**
	3	**79.7**	33.4	**117.1**	27.9	4.553	**p < 0.001**
	6	**52.6**	30.5	**108.0**	38.7	5.569	**p < 0.001**
	12	**22.1**	14.4	**41.3**	24.4	3.995	**p < 0.001**
	18	**6.42**	5.40	**16.2**	11.5	4.189	**p < 0.001**
Day testing with glare	1.5	**40.2**	15.8	**64.3**	23.6	4.392	**p < 0.001**
	3	**84.9**	30.9	**127.4**	26.1	5.064	**p < 0.001**
	6	**59.9**	39.1	**112.3**	37.9	5.201	**p < 0.001**
	12	**25.9**	20.0	**47.0**	25.8	4.028	**p < 0.001**
	18	**7.15**	6.01	**17.5**	12.5	3.971	**p < 0.001**

*MS* multiple sclerosis, *x* mean, *SD* standard deviation

**Table 2:** Statistics of contrast sensitivity measurements, in daylight/night conditions, with and without glare, in patients after optic neuritis

Examination	Spatial frequency	Calculated contrast parameters					
		min	max	x	Me	SD	v(%)
Night testing without glare	1.5	13	50	**31.9**	36	12.1	37.8
	3	10	160	**70.4**	57	32.9	46.7
	6	0	90	**34.3**	33	23.4	68.4
	12	0	22	**8.45**	8	7.52	88.9
	18	0	12	**0.91**	0	2.50	275.5
Night testing with glare	1.5	18	71	**39.9**	36	19.2	48.1
	3	10	160	**72.1**	57	34.2	47.4
	6	0	128	**35.9**	33	27.7	77.0
	12	0	30	**9.79**	11	8.34	85.2
	18	0	8	**0.61**	0	1.97	324.6
Day testing without glare	1.5	13	71	**28.6**	25	12.7	44.2
	3	29	160	**79.7**	80	33.4	41.9
	6	0	128	**52.6**	45	30.5	57.9
	12	0	60	**22.1**	22	14.4	65.3
	18	0	17	**6.42**	8	5.40	84.0
Day testing with glare	1.5	18	71	**40.2**	36	15.8	39.3
	3	29	160	**84.9**	80	30.9	36.4
	6	0	180	**59.9**	45	39.1	65.3
	12	0	85	**25.9**	22	20.0	77.3
	18	0	17	**7.15**	8	6.01	84.0

*min* minimum, *max* maximum, *x* mean *Me* median, *SD* standard deviation, *v* variance

Our study also confirms that a reduction of contrast sensitivity is observable in all spatial frequencies—low, medium, and high.

The results of OCT measurements in a group of patients with multiple sclerosis show that retinal nerve fiber layer thickness (RNFL) was between 56 and 122 μm (mean 98.3; standard deviation 16.2). All patients with MS, except one, had RNFL thickness above 75 μm. RNFL thickness in a group of healthy control subjects was from 78 to 126 μm (mean 100.9; standard deviation 14). There were no statistically significant differences between mean RNFL thickness in both groups.

## Discussion

Decreased contrast sensitivity is noticeable in low, intermediate, and high spatial frequencies in patients with an acute inflammatory phase, in patients after neuritis, and in patients with multiple sclerosis without a history of optic neuritis [23]. In patients who haven't had optic neuritis in the course of multiple sclerosis, contrast sensitivity was decreased in intermediate and high spatial frequencies (12–18 cpd) [26].

In patients with optic neuritis, an increase in visual acuity can be observed after 2–4 weeks, to the level of 0.6 or better. Nevertheless, color perception and contrast sensitivity are reduced. In about 10 % of patients, chronic optic neuritis develops, with no remissions and with a constant and gradual decrease of visual acuity [1, 20, 27–31].

Degeneration of the optic nerve axons causes the disturbance of retinal nerve fiber layer thickness, which can be documented with OCT. What's more, OCT allows an estimate of the relationship between RNFL thickness and visual function after optic neuritis. It is thought that the first changes in RNFL thickness can be seen in the temporal quadrant, as early as 2 months after the inflammatory episode. The decrease in RNFL thickness in eyes after optic neuritis is visible for up to 24 months; during the first 6 months the changes increase, and later on, they stabilize [32].

Regression analysis helped to determine the minimal RNFL thickness (amounting to 75 μm) that allowed for the recovery of vision after optic neuritis [32, 33]. Retinal nerve fiber thickness was found to correlate with visual acuity, contrast sensitivity, color vision, and visual field defects [20].

There was a significant decrease of RNFL thickness in eyes after optic neuritis caused by multiple sclerosis, in comparison with the fellow eye and with healthy subjects. In addition, an RNFL below 75 μm was significantly correlated with visual field defects. It has been observed that despite the fact that visual acuity returned to normal after optic neuritis, patients still complain of subjective decreased vision, which may result from decreased contrast sensitivity or visual field defects [21, 31, 34].

There are few characteristic types of changes in contrast sensitivity in patients who had optic neuritis in multiple sclerosis: (1) decreased contrast sensitivity in all spatial frequencies; (2) decreased contrast sensitivity in intermediate and high spatial frequencies; (3) decreased contrast sensitivity in only intermediate spatial frequencies; (4) decreased contrast sensitivity in only low spatial frequencies; and (5) unchanged contrast sensitivity [31]. The last two types mentioned are the least frequently encountered.

In examinations evaluating color vision and contrast sensitivity in patients with multiple sclerosis, it has been observed that defects may be selective or generalized. Dain et al. [29] proved the existence of two outcomes among patients after optic neuritis. The first group had a selective defect of color vision in the red-green axis, as well as selective impairment of contrast sensitivity in high spatial frequencies. The second group had a generalized defect of color vision, concerning both the red-green axis and blue-yellow axis, as well as a generalized impairment of contrast sensitivity irrespective of spatial frequency. The authors conclude that this phenomenon may result from the demyelinative damage to the optic nerve, which may be either selective and include mainly small axons, or that it may be generalized and include both small and large axons.

Rekas et al. [35] draw similar conclusions regarding selective and non-selective damage to the optic nerve in patients with multiple sclerosis. They found that one group of patients had decreased contrast sensitivity at 18 cpd, as well as defective blue-color perception, whereas the second group of patients had decreased contrast sensitivity at 12 cpd, as well as defective red-color perception. In the third (and the largest) group of patients, there was a generalized (non-selective) defect in color perception and generalized decreased contrast sensitivity, with a predilection for higher spatial frequencies.

The results of our study are consistent with the results of previously published articles. However, we have performed a full evaluation of contrast sensitivity in photopic conditions (with and without glare), as well as in mesopic conditions (with and without glare) in patients with MS, which revealed a non-selective significant reduction of contrast sensitivity. Furthermore, this study emphasizes the important role of contrastometry in patients with MS, as a method that can identify the deficits of visual function that otherwise go undetected by a simple visual acuity measurement.

## Conclusions

Contrast sensitivity in patients with multiple sclerosis who also suffer from optic neuritis is significantly reduced in all spatial frequencies, both in photopic conditions (with and without glare) and in mesopic conditions (with and without glare). This may explain patients' complaints regarding their quality of vision, despite having good visual acuity.

Contrastometry is a useful basis for examination in this context, providing additional information regarding the patient's quality of vision.
